# Possible Racial Disparities in the Diagnosis of Myalgic Encephalomyelitis/Chronic Fatigue Syndrome (ME/CFS)

**DOI:** 10.3390/ijerph22020280

**Published:** 2025-02-14

**Authors:** Chloe Lisette Jones, Jarred Younger

**Affiliations:** Department of Psychology, University of Alabama at Birmingham, 1300 University Blvd, Birmingham, AL 35294, USA; youngerlab@uab.edu

**Keywords:** bias, social determinants of health, minority, long COVID, inequality

## Abstract

Myalgic encephalomyelitis (ME/CFS) a chronic, disabling illness with no established etiopathology. It has been indicated in some population-based studies that Black and ethnic minority populations are underdiagnosed with ME/CFS. The aims of the present study were to (1) identify the agreement between receiving an ME/CFS diagnosis and meeting diagnostic criteria, (2) identify the demographic characteristics associated with receiving a diagnosis, and (3) explore patient satisfaction with healthcare. Self-reported medical history and symptoms were collected via online survey from respondents with and without fatigue. The agreement between self-reporting an ME/CFS diagnosis and meeting the Center for Disease Control’s (CDC) ME/CFS criteria or Institute of Medicine (IOM) criteria was assessed with Cohen’s kappa. Patient characteristics predicting a physician diagnosis were analyzed with logistic regression. Associations between diagnosis, demographics, and healthcare satisfaction were assessed with chi-square tests of independence. There were 1110 responses. The agreement between meeting ME/CFS criteria and reporting an ME/CFS diagnosis was fair (CDC: κ = 0.29; SE = 0.02; IOM: κ = 0.28, SE = 0.03). White respondents had 2.94 greater odds of being diagnosed with ME/CFS than non-White respondents. Having an ME/CFS diagnosis was associated with dissatisfaction with healthcare (χ^2^ (3, N = 1063) = 14.17, *p* = 0.003). The findings suggest racial disparities in the diagnostic processes for ME/CFS.

## 1. Introduction

Myalgic encephalomyelitis/chronic fatigue syndrome (ME/CFS) is a long-term, disabling, multisystem illness. The illness is predominantly theorized to be a post-infectious neuroinflammatory syndrome [1,2]; however, microbiome disturbances [3,4], mitochondrial dysfunction [5,6], and vascular mechanisms [7,8] have also been proposed. The most common symptoms include severe fatigue and sleep problems, cognitive dysfunction, pain, dizziness, and post-exertional malaise (PEM), which is the worsening of symptoms after exertion or activity [9]. The Center for Disease Control (CDC) 1994 case criteria for ME/CFS, often referred to as the Fukuda case criteria, require persistent or relapsing fatigue for six months or greater that is not alleviated by rest, and at least four of eight secondary symptoms: cognitive symptoms, sore throat, tender lymph nodes, muscle pain, joint pain without swelling or redness, headaches, unrefreshing sleep, and PEM. In 2015, the Institute of Medicine (IOM) published ME/CFS diagnostic criteria that further specified that fatigue, PEM, and unrefreshing sleep must be present for diagnosis along with the presence of cognitive impairment and/or orthostatic intolerance [10].

An ME/CFS diagnosis relies on differential diagnosis exclusion and patient reporting of symptoms. The cardinal symptom of chronic fatigue is nonspecific and may be induced by a variety of conditions and disease processes, such as endocrine dysfunction, renal dysfunction, hepatic dysfunction, blood sugar dysregulation, psychological conditions, neurological conditions, hematological conditions, infections, deficiencies, cancers, gastrointestinal diseases, respiratory issues, autoimmune conditions, sleep disorders, chronic pain, cardiovascular conditions, allergies, stress, and more. Because it relies on clinical judgement and patient self-reporting, diagnosis of ME/CFS may be particularly susceptible to bias.

It has been demonstrated that clinicians are not confident in diagnosing ME/CFS [11,12,13] compared to other disorders, but the exact degree of inconsistency in ME/CFS diagnosis is unknown. In a study of fibromyalgia (FM), which has significant overlap with ME/CFS, it was found that 75% of patients who reported a clinical diagnosis of FM by a physician did not actually meet FM criteria [14]. Instead of meeting case definition criteria, the best predictors of receiving a FM diagnosis were being White, female, and married. However, none of these demographic variables predicted meeting case criteria. The researchers further concluded that if a patient met FM criteria, but was not White, married, and female, there was little chance of receiving a diagnosis [14]. Another study found that physicians failed to identify half of FM patients who did meet criteria [15]. It is unknown if the agreement between diagnosis and criteria in ME/CFS is as poor as in FM.

Diagnosis of ME/CFS is lower in Black and ethnic minority populations [16]. Direct comparison of symptomatology between White and Black individuals with ME/CFS found little to no differences, suggesting it is not symptom presentation that may be responsible for these discrepancies in diagnosis. A narrative synthesis and meta-analysis of ME/CFS patient samples identified that population-based studies were more likely to identify higher risks of ME/CFS in ethnic minority groups, with some findings suggesting a greater likelihood of ME/CFS in ethnic minority groups, though this is not consistently reported in all population based studies [17]. A community-based sample in the Chicago area of pediatric ME/CFS identified even higher prevalence in African American and Latinx youth than Caucasian youth [18]. This pattern has not been observed in physician-based referral prevalence studies, suggesting access to care influences the ME/CFS population demographics.

When individuals are diagnosed with ME/CFS, they face few treatment options. A Norway-based sample of ME/CFS patients interviewed about their healthcare experiences often were confronted with a lack of healthcare services, delay of services, and provision of services that were inappropriate or poorly adapted to their level of functioning [19]. The majority of ME/CFS patients have not seen ME/CFS specialists due to the relatively low number of specialists, their geographic restrictions, and sometimes prohibitive costs [20]. Although it is established that ME/CFS patients face substantial barriers to care, it is unknown how healthcare experiences may differ from those with other chronic conditions who may also be faced with few treatment options. Because of the stigma experienced by patients with ME/CFS [13,21,22], the diagnosis itself may result in barriers to treatment and poor satisfaction with care that cannot be entirely explained by the difficulty in treating the symptoms. It is unknown to what degree the diagnosis of ME/CFS itself, as opposed to the symptoms of ME/CFS, results in poor healthcare experiences.

The aim of this survey-based, observational study was to identify the agreement between reporting an ME/CFS diagnosis and meeting diagnostic criteria, and to identify if there are demographic characteristics associated with receiving an ME/CFS diagnosis. A secondary aim was to explore ME/CFS participants’ satisfaction with their healthcare providers and compared to those with other health problems. We also explored whether satisfaction with care and barriers to care were associated with ME/CFS symptomatology (i.e., meeting criteria), with having an ME/CFS diagnosis, and/or with certain demographic characteristics.

## 2. Materials and Methods

### 2.1. Recruitment

An online survey was utilized to obtain self-reported symptoms, medical histories, participant characteristics, and validated symptom assessments. The survey was created with Qualtrics XM software (Qualtrics, Provo, UT, USA). We collected responses from individuals with diverse, chronic health conditions, although recruitment efforts were focused on individuals with chronic pain and fatigue. The survey was distributed via patient forums, email lists of chronic health organizations, and social media postings, with consent of the administrative bodies of these organizations/forums. Although the survey was distributed across the US and to other countries, the laboratory was based at the University of Alabama at Birmingham (UAB). The survey was also distributed to individuals who participated in our laboratory’s prior research studies and who indicated they would like to be contacted for future research. Healthy individuals without chronic conditions, or with mild symptoms of fatigue and pain, were also recruited. The remote design allowed participants who lived in rural locations or had issues with transportation to participate.

### 2.2. Study Procedures

Informed consent was obtained at the beginning of the survey, and participants were asked to confirm English proficiency and age of 18 years or older. Participants were required to complete a captcha response for internet bot detection. Those indicating they were younger than 18 and/or not proficient in the English language were redirected to the termination of the survey. Twenty respondents were randomly selected with a random number generator to receive a USD 50 e-gift card to their provided email address. The entire survey was estimated to take a total of 1–2 h to complete. Due to display logic, total completion time was variable. Participants did not have to complete the survey within one sitting. Throughout the survey, participants were informed of their progress and estimated time to completion.

### 2.3. Measures

#### 2.3.1. ME/CFS Symptoms

Case criteria from Fukuda et al. [9] and the IOM [10] were included in the survey to provide a comprehensive coverage of ME/CFS symptoms. The survey also included content derived from the NIH Common Data Elements for ME/CFS (https://www.commondataelements.ninds.nih.gov/ (accessed on 12 February 2025)) to improve synthesizability across studies, and to capture all domains of interest.

Responses were defined as meeting Fukuda criteria and/or IOM criteria using the DePaul Symptom Scale (DSQ-2) and Short Form-36 (SF-36) [23] to assess for the presence of symptoms and a reduction in functioning, with the same method as described in Bedree et al. [24] and the supplementary scoring methods provided by Jason and Sunnquist [25]. The DSQ-2 probes frequency and severity of ME/CFS symptoms on a 5-point Likert scale over the past 6 months. As in Bedree et al. [24], each symptom was converted into a composite score, ranging from 0 to 100, by averaging the frequency and severity score for that symptom multiplied by 25. A symptom was determined to be present if rated by a respondent to be present at least half the time and of at least moderate severity. Those who listed comorbid exclusionary conditions [26] were not considered positive ME/CFS cases.

The SF-36 [23] is a reliable and frequently cited measure that assesses general health and is not specific to any single condition, and is therefore useful in a diverse sample. To assess for a substantial reduction in functioning, participants’ scores on two out of the following three domains had to be lower than the respective cut-offs: lower than 50 for the physical role limitations domain, lower than 62.5 for the social functioning domain, and lower than 35 on the vitality/energy domain. Items from the DSQ-2 and DePaul PEM Questionnaire [27] were listed in their original form.

#### 2.3.2. General Medical History

To query general health status, participants were asked whether they considered themselves to have “significant health problems or medical conditions”, considered themselves to be “mostly healthy with some minor medical conditions or health problems”, or considered themselves “healthy, with no major medical conditions or health problems”. Reported medical history of all current and past conditions, illnesses, all past surgeries and hospitalizations, infectious histories, known immunizations, injuries, allergies, and sensitivities was collected. Individuals were provided with a checklist of common medical conditions including ME/CFS and asked to select which conditions they had been diagnosed with. They were also then provided with free-text items to list all medical diagnoses they have received.

#### 2.3.3. Satisfaction with Care

Respondents who indicated that they had minor or major health conditions were asked “have you ever seen a doctor or health professional about your health problem?” with the following response options: (1) “Yes”, (2) “No, but I have tried”, or (3) “No, and I do not plan to”. They were also asked, “Do you currently have a doctor overseeing your health problem?” with the following response options: (1) “No, and I do not want one”, (2) “No, but I wish I did”, (3) “Yes, and I am unsatisfied with my doctor/health professional”, or (4) “Yes, and I am satisfied with my doctor/health professional”.

#### 2.3.4. Demographic Information

Demographic information collected can be seen in Table 1. For the purposes of this study, participants were asked their sex assigned at birth, gender, ethnicity, race, education, country of residence, and yearly household income.

### 2.4. Analysis

#### 2.4.1. General Screening Procedures

RelevantID technology (Imperium, Shelton, CT, USA) is utilized by the Qualtrics XM platform (Provo, UT, USA) to detect possible fraudulent and duplicate responses with multiple data points including geo-location, time, language, IP address, and captcha response quality. Responses flagged as potential duplicate or fraudulent responses were removed from the dataset. The Conscientious Responders Scale (CRS) [28] was used to detect random responding. This five-item scale, dispersed throughout the survey, directs participants how to respond and correctly classifies random responders more than 93% of the time. As recommended by the authors of the CRS, respondents with a score of less than 3 on the scale were considered random responders and excluded from the dataset [28].

#### 2.4.2. Statistical Analysis

Only participants who had complete data for ME/CFS criteria and complete data for physician diagnoses were included. Cronbach’s alpha was calculated for the DSQ-2 and SF-36 to determine internal consistency in this sample.

To assess the agreement between reporting a clinical ME/CFS diagnosis and meeting ME/CFS diagnostic criteria, kappa analysis was employed. SPSS version 28 (IBM Corp., Armonk, NY, USA) was used to calculate Cohen’s kappa through the Crosstabs procedure. Cohen’s kappa of 0.00 or less indicates poor agreement; 0.00–0.20 is slight; 0.21–0.40 is fair; 0.41–0.60 is moderate; 0.61–0.80 is substantial; and 0.81–1.00 is almost perfect agreement [29]. Binomial logistic regression was performed to assess which demographic variables best predicted receiving a diagnosis. Individuals without a diagnosis of ME/CFS served as the reference group.

To explore satisfaction with care, chi-square tests of independence were performed to assess the association between having an ME/CFS diagnosis, reporting having a doctor or health professional overseeing their health problem, and being satisfied or unsatisfied with their doctor or other health professional.

Missing data were not imputed or estimated to avoid introducing bias in the dataset, which was exploratory by nature. Incomplete data were analyzed with pairwise deletion. Whether or not missing data were significantly different from included data on the variables of age, gender, race, and health status was assessed using one-way ANOVA and Pearson chi-square tests.

Due to a low frequency of responses reporting a gender other than man or woman, responses were grouped into ‘male’, ‘female’, and ‘other’. Racial categories were also grouped into ‘White’ and ‘non-White’ due to relatively low frequencies of responses across non-White racial categories.

## 3. Results

### 3.1. Data Screening Procedures

#### 3.1.1. Missingness

Responses were collected between October 2022 and May of 2023, for a total number of 2731 responses. Participants who completed 5% or less of the survey were removed due to the high degree of missing data (*n* = 612). In this sample, 1303 respondents indicated that they had received a diagnosis of ME/CFS (68%), while 566 did not (30%). Forty-two respondents had missing data for this item and were excluded from the present analysis (total *n =* 1869). Responses were then classified into whether CDC criteria were fulfilled or not and whether IOM criteria were fulfilled or not. Responses with missing data for any of the requisite items to determine ME/CFS criteria were treated as missing and excluded (*n* = 782), resulting in a total of 1110 responses included in the present analyses for CDC criteria and 1036 responses for IOM criteria. Excluded cases were not significantly different for the factors of race (χ^2^(1) = 0.236, *p* = 0.627), gender (χ^2^(2) = 0.68, *p* = 0.71), age (F(1, 1883) = 0.21, *p* = 0.65), or self-reported general health status (χ^2^(2) = 1.13, *p* = 0.29).

#### 3.1.2. Fraud Detection

Based on the recommendations provided by Qualtrics, 32 responses were identified as possible bots based on a poor captcha score (less than 0.5); 108 responses were identified as possible duplicate submissions (duplicate score of 75 or greater); 113 rows were identified as being possibly fraudulent (fraud score of 30 or greater).

As participants varied in their progress through the survey, not all respondents were exposed to all five CRS items. The original CRS scoring criteria recommends a cutoff of fewer than three out of five CRS items correct. Therefore, respondents scoring lower than 60% on the CRS scale were removed (*n* = 55).

As a result of these screening procedures, 1908 responses were maintained in the dataset (70% of the full sample). Pearson chi-square and one-way ANOVA tests were employed to assess whether the excluded cases varied from the included cases on several participant characteristics. The two groups did not differ by age (F(1, 2310) = 1.22, *p* = 0.27), or race (χ^2^(1) = 2.47, *p* = 0.12); however, the included sample had a lower frequency of healthy responses (χ^2^(2) = 8.89, *p* = 0.003), and a lower frequency of responses from males (χ^2^(2) = 27.39, *p* < 0.001). This indicates that responses indicating being healthy or being male were more likely to be fraudulent, duplicate, or highly sparse.

### 3.2. Sample Characteristics

The majority of respondents identified themselves as having “significant health problems or medical conditions” (*n* = 1528, 81%), while 16% reported being “mostly healthy with some minor medical conditions or health problems” (*n* = 304), and 3% of the sample (*n* = 61) reported being “healthy, with no major medical conditions or health problems”. Participants reported a wide range of medical conditions. ME/CFS was the most well-represented illness in the sample; 1303 of the respondents reported an ME/CFS diagnosis (68%). Other conditions with notable representation included FM (*n* = 859, 45%), irritable bowel syndrome (IBS) (*n* = 805, 42%), and postural orthostatic tachycardia syndrome (POTS) (*n* = 624, 33%). Many respondents also reported gynecological issues (*n* = 655, 34%), other bowel/stomach problems (*n* = 554, 29%), a psychiatric disorder (*n* = 586, 31%), thyroid problems (*n* = 522, 27%), and neurological problems (*n* = 342, 18%).

The sample was majority female (*n* = 1594, 84%) with a mean age of 53.04 (SD = 14.13), a yearly gross income of less than USD 70,000 (*n* = 519, 55%), and a 4-year degree or higher (*n* = 732, 69%). Demographic sample characteristics can be seen in Table 1. Among this sample, 56.91% (*n* = 597) reported living in the United States. The next most common countries of residence were the United Kingdom (*n* = 104), Canada (*n* = 77), Australia (*n* = 56), and Norway (*n* = 49).

### 3.3. Agreement Between Meeting Criteria and Reporting a Diagnosis

**Figure 1:**
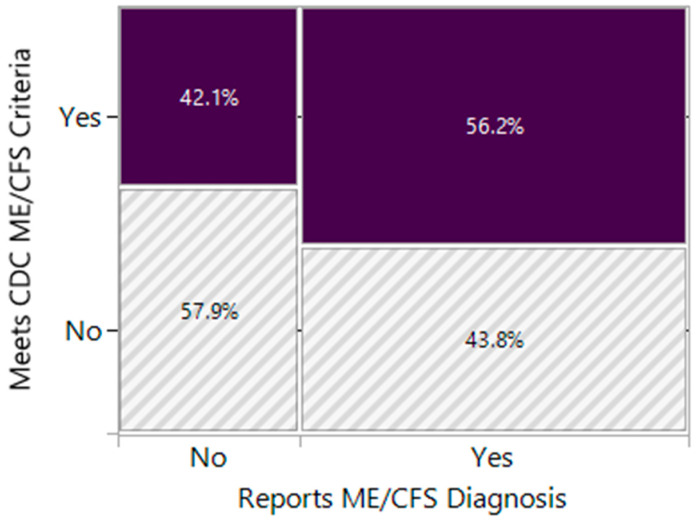
Mosaic plot of ME/CFS diagnosis by CDC ME/CFS criteria. The area of the rectangles is proportional to the number of observations in that category. The width of the columns is proportional to the number of responses in each category of the x-axis (reports an ME/CFS diagnosis). The vertical length of the bars is proportional to the number of responses in each category of the y-axis (meets CDC ME/CFS criteria) within each x-axis category (reports ME/CFS diagnosis).

The DSQ-2 (α = 0.97) and SF-36 (α = 0.88) were found to be internally consistent. To assess agreement between meeting ME/CFS criteria and reporting an ME/CFS diagnosis, the Cohen’s kappa agreement statistic was computed for both CDC and IOM criteria. Results of the contingency analysis revealed that the degree of agreement between receiving a diagnosis and meeting CDC criteria was fair (κ = 0.29, SE = 0.025), but significantly different from zero (*p* < 0.001). Bowker–McNemar’s test indicated that the contingency table was not symmetric (χ^2^(1) = 159.77, *p* < 0.001). The agreement between meeting IOM criteria and reporting a diagnosis was also fair (κ = 0.28, SE = 0.026) and significantly different from zero (*p* < 0.001). Bowker–McNemar’s test indicated that the contingency table was not symmetric (χ^2^(1) = 169.81, *p* < 0.001). In this sample, 68% reported an ME/CFS diagnosis, 45% of the respondents met CDC criteria for ME/CFS (Table 2), and 42% met IOM criteria. Of those reporting an ME/CFS diagnosis, 44% were not found to meet CDC criteria (Figure 1), and 43% were found not to meet IOM criteria. Of those who met IOM criteria, 15% did not report a diagnosis of ME/CFS, and of those who met CDC criteria, 15% did not report a diagnosis of ME/CFS. Of these individuals who fulfilled criteria but did not report a diagnosis, 61% had a diagnosis of FM.

### 3.4. Patient Features That Predict Reporting an ME/CFS Diagnosis

The sample comprised 946 women, 155 men, and 26 categorized as ‘other’. There were 1065 White respondents, while 58 were non-White, and 28 did not report their racial identity. The full model (−2LL of 1362.62) was significantly better than the null model with no predictors (−2LL = 1381.42, χ^2^(3) = 18.80, *p* < 0.001). The respondent’s race was a significant predictor of reporting an ME/CFS diagnosis (Wald (1) = 14.329, *p* < 0.001). White respondents had 2.94 greater odds of being diagnosed with ME/CFS than non-White respondents. Gender was not a significant predictor (Wald (2) = 3.18, *p* = 0.20).

### 3.5. Satisfaction with Care

In this sample, 98.35% (*n* = 1015) of respondents with health conditions reported having seen a doctor or health professional for their health problem. Those with ME/CFS were more likely than expected to report not having a doctor or health professional, but wishing they had one (10.54%, *n =* 112), and more likely than expected to report being unsatisfied with their doctor or health professional (43.78%, *n =* 317). The overall chi-square test of independence was significant (χ^2^(3, *n* = 1063) = 14.170, *p* = 0.003), indicating the relative frequencies of response options were different between those with and without a diagnosis of ME/CFS. When comparing responses between those who met Fukuda criteria and those who did not, regardless of formal diagnosis, the overall chi-square test of independence was not significant (χ^2^(3, N = 1063) = 3.071, *p* = 0.381).

A chi-square test of independence was performed comparing response options and self-reported race. Response options were significantly different between White and non-White individuals (χ^2^(3, N = 1058) = 14.132, *p* = 0.003). The greatest deviation between expected and observed frequencies was the greater than expected number of non-White individuals reporting they did not have a doctor overseeing their health problem and did not want one. This accounted for most of the overall chi-square statistic (12.750). The overall frequency of this response was still low overall in the sample (*n* = 25). Although non-White individuals comprised only 4.63% of the overall sample, they accounted for 20.00% of those who stated they did not have a doctor overseeing their health problem and did not want one.

## 4. Discussion

Approximately half of the participants reporting an ME/CFS diagnosis met CDC criteria (56%) or IOM criteria (48%) and the agreement between reporting a diagnosis and meeting diagnostic criteria was found to be only fair. These results are consistent with what has been observed in diagnoses and self-reported symptoms in FM [7].

There are several possible reasons for the relatively large proportion of individuals reporting an ME/CFS diagnosis, but not meeting criteria. One possibility is that several individuals may have managed many of their symptoms to the degree that they no longer fulfil criteria, though rates of full remission are generally low in this population [24,25,26]. Second, participants’ providers may have used diagnostic criteria other than the CDC Fukuda et al. criteria or IOM criteria. This might be particularly relevant for patients and providers outside the United States, who may use other criteria such as the International Consensus Criteria [27]. CDC criteria, however, are thought to encompass a broader sample than other criteria, and therefore it is considered relatively unlikely that many participants who did not fulfil CDC criteria fill other ME/CFS case criteria. Ultimately, if different case criteria were responsible for the large discrepancy, that indicates what may be perceived as slight divergences among various diagnostic criteria in practice can result in significantly different patient populations. Despite these possible explanations, the present results still suggest diagnostic processes for ME/CFS are biased or unstandardized.

There were a lower number of participants meeting IOM or CDC ME/CFS criteria who did not report a diagnosis. The majority of these individuals (61%) reported an FM diagnosis. If the majority of a patient’s symptoms can be explained by FM, an additional ME/CFS diagnosis may not be deemed as warranted by a clinician. Diagnosis of ME/CFS may also have been complicated by the varying diagnostic codes historically. Furthermore, physicians may favor an FM diagnosis over an ME/CFS diagnosis as there exist FDA-approved treatment options for FM, though this has yet to be explicitly investigated.

Preliminary analyses explored whether sociodemographic factors influence diagnostic processes, as has been seen in FM [7]. The present results revealed that White participants had 2.94 greater odds of being diagnosed with ME/CFS. Although these results are consistent with prior research, this finding should be interpreted with caution as the overall number of participants who were not White in this sample was low (*n* = 58). As this study recruited one of the largest ME/CFS convenience samples, it is unknown whether the demographics of this sample were biased or accurately reflective of the ME/CFS population. Further research into this topic with a more balanced and representative sample is encouraged. As English language proficiency was required for participation in this study, the results are not generalizable to those of other language groups, or those with poor literacy. Clinically, language barriers likely impact individuals’ access to diagnosis and care, though this was not examined in the present study.

There are several proposed mechanisms for the racial disparities in the diagnosis of ME/CFS that future research can investigate. There is the possibility that ME/CFS is more likely to affect those of European decent or those from higher latitudes, such as in multiple sclerosis [28]. Another possibility is that patients who receive ME/CFS diagnoses reflect those with greater self-advocacy and persistence in healthcare settings, as diagnosis can often take several years and multiple providers [29]. Standard diagnostic tests tend to yield normal results in ME/CFS, so clinicians are more reliant on their judgement of a clinical presentation. Therefore, clinicians may be more likely to identify ME/CFS in White women as it is more consistent with the prototypical or exemplar cases. This possibility may be particularly pertinent for ME/CFS given its initial historical conceptualization as ‘yuppie flu’ [30]. These suggestions are potential avenues for future research to confirm whether biased diagnostic processes exist in this population.

Diagnosis of ME/CFS is further complicated by its high degree of comorbidity. A recent population-based study in Spain identified that more than 80% of patients with ME/CFS had comorbid conditions [31], while a Canadian Community health survey found 65.2% to have three or more comorbidities [32]. The clinical overlap between ME/CFS and FM is particularly strong, with 34–75% of those with ME/CFS meeting FM criteria [33,34]. Conversely, approximately 42–70% of those with FM meet ME/CFS criteria [35,36]. The conditions overlap so significantly that some individuals given a diagnosis of ME/CFS by one clinician may be given a diagnosis of FM by another clinician. When both disorders were included in one study, latent class analysis did not assign FM and ME/CFS patients into different groups [37]. In a more recent study, there were no differences in pain, fatigue, or quality of life between those with ME/CFS and those with both ME/CFS and FM [38]. In the same study, no differences in pain, fatigue, or quality of life were found between those with ME/CFS, or those with ME/CFS and FM. It is therefore of interest to determine which patient characteristics warrant a diagnosis of ME/CFS over another candidate diagnosis.

More than 10% of individuals with ME/CFS reported not having a doctor overseeing their health problem but wanting one, and approximately 45% of those with ME/CFS reported being unsatisfied with their doctor/health care professional. Interestingly, this pattern was not replicated when comparing those who met ME/CFS criteria and those who did not. This suggests that barriers to care and satisfaction with care may be affected directly by the ME/CFS diagnosis itself, rather than the nature of the symptoms. As the majority of participants in this sample presented with comorbidities, reflective of the ME/CFS population, future research should investigate the impact of having an ME/CFS diagnosis on the overall provision of healthcare services to an individual. Specific mechanisms (e.g., severity of presentation, demographics, stigma in healthcare settings, comorbid conditions, etc.) underlying the association between an ME/CFS diagnosis and healthcare outcomes should be further studied.

Given the relatively recent identification of ME/CFS in the ICD-10, outreach efforts to educate community health professionals about ME/CFS may be particularly timely and impactful in addressing barriers to treatment. A recent educational webinar on ME/CFS was found to improve healthcare professionals’ knowledge of the illness, who on average reported low-to-moderate confidence in diagnosing the condition [39]. By improving providers’ knowledge and reducing the typically lengthy diagnostic process for ME/CFS, satisfaction with care may also improve in this population. Regional ME/CFS centers could also potentially aid in diagnosis and foster knowledge dissemination to local practitioners.

It was also observed in this study that individuals who were non-White were more likely to report not having a doctor and not wanting a doctor overseeing their health problem than White individuals. It is well known that racial and ethnic minorities have greater barriers to care and more medical mistrust than their White counterparts [40,41,42]. These findings, together with prior literature, indicate that those who identify as non-White and also have ME/CFS reflect a particularly vulnerable intersection regarding barriers to care and poor healthcare satisfaction. Non-White individuals with ME/CFS are a critically understudied population, and due to their greater risk of poor health outcomes, and poor healthcare provision, warrant greater focus in clinical research.

Education and training has been the most common strategy used to address racial disparities in healthcare [43]. The present findings demonstrate that future educational interventions for ME/CFS should include information about the racial and ethnic disparities that exist in this population. Learning objectives should aim to improve providers’ confidence in diagnosing ME/CFS generally, but also specifically aim to improve their confidence in recognizing ME/CFS in a diverse range of individuals.

## 5. Conclusions

Among a large sample of individuals with fatigue, agreement between meeting ME/CFS criteria and reporting an ME/CFS diagnosis was only fair. White respondents had significantly greater odds of being diagnosed with ME/CFS than non-White respondents. These findings suggest racial disparities in the diagnostic processes for ME/CFS. Furthermore, diagnosis of ME/CFS, but not meeting ME/CFS criteria, was associated with poorer satisfaction with healthcare, suggesting the diagnosis itself may present barriers in the management of symptoms.

## Figures and Tables

**Table 1:** Demographic characteristics of the study sample.

Demographics	N	% of Total
Sex Assigned at Birth		
Male	254	13.44%
Female	1635	86.51%
Intersex	1	0.05%
Gender		
Male	255	13.47%
Female	1594	84.20%
Non-binary/Third Gender	32	1.69%
Prefer Not to Say	5	0.26%
Other	7	0.37%
Ethnicity		
Hispanic	53	2.81%
Non-Hispanic	1834	97.19%
Race		
White	1787	94.90%
Black, African American	41	2.18%
American Indian or Alaskan Native	28	1.49%
Asian	32	1.70%
Native Hawaiian or Pacific Islander	7	0.37%
Other	71	3.77%
Education		
Less than High School Degree	16	1.50%
High School Diploma or Equivalent	58	5.44%
Some College	155	14.54%
Associate Degree (2-year)	105	9.85%
Bachelor’s Degree (4-year)	353	33.11%
Master’s Degree	265	24.86%
Doctoral or Professional Degree	114	10.69%
Yearly Gross Household Income		
Less than USD 10,000	68	7.16%
USD 10,000 to USD 19,999	90	9.47%
USD 20,000 to USD 29,999	85	8.95%
USD 30,000 to USD 39,999	73	7.68%
USD 40,000 to USD 49,999	71	7.47%
USD 50,000 to USD 59,999	71	7.47%
USD 60,000 to USD 69,999	61	6.42%
USD 70,000 to USD 79,999	57	6.00%
USD 80,000 to USD 89,999	39	4.11%
USD 90,000 to USD 99,999	70	7.37%
USD 100,000 to USD 149,999	136	14.32%
USD 150,000 or more	129	13.58%

**Table 2:** Frequencies of reported ME/CFS diagnoses and meeting CDC ME/CFS criteria.

		Reported Diagnosis		
		No	Yes	Total
Met CDC Criteria	Yes	76	425	501
	No	278	331	609
	Total	354	756	1110

## Data Availability

The data supporting the conclusions of this article will be made available by the authors on request.
